# Influence of Density on Foam Collapse under Burning

**DOI:** 10.3390/polym13010013

**Published:** 2020-12-22

**Authors:** Abdoul Fayçal Baguian, Salifou Koucka Ouiminga, Claire Longuet, Anne-Sophie Caro-Bretelle, Stéphane Corn, Antoine Bere, Rodolphe Sonnier

**Affiliations:** 1 Laboratoire de Physique et de Chimie de l’Environnement, Université Joseph KI-ZERBO, Ouagadougou 03 BP 7021, Burkina Faso; bag_fay@yahoo.fr (A.F.B.); salif0477@yahoo.com (S.K.O.); berebiya@yahoo.fr (A.B.); 2 IMT—Mines Ales, Polymers Hybrids and Composites (PCH), 6 Avenue De Clavières, F-30319 Alès CEDEX, France; claire.longuet@mines-ales.fr; 3 LMGC, IMT Mines Ales, Université Montpellier, CNRS, F-30319 Alès CEDEX, France; anne-sophie.caro@mines-ales.fr (A.-S.C.-B.); stephane.corn@mines-ales.fr (S.C.)

**Keywords:** polyurethane foam, fire behaviour, collapse

## Abstract

The fire behaviour of flexible polyurethane foams was studied using a cone calorimeter, with a special emphasis on the collapse step. Only one peak of heat release rate, ranging from 200 to 450 kW/m^2^, is observed for thin foams, depending on the foam density and the heat flux. On the contrary, heat release rate (HRR) curves exhibit two peaks for 10 cm-thick foams, the second one corresponding to the pool fire formed after foam collapse. In all cases, the collapse occurs at a constant rate through the whole thickness. The rate of the recession of the front was calculated using digital and infrared cameras. Interestingly, its value is relatively constant whatever the heat flux (especially between 25 and 35 kW/m^2^), probably because of the very low heat conductivity preventing heat transfer through the thickness. The rate increases for the lightest foam but the fraction of burnt polymer during collapse is constant. Therefore, the pool fire is more intense for the densest foam. A simple macroscopic model taking into account only the heat transfer into the foam leads to much lower front recession rates, evidencing that the collapse is piloted by the cell walls’ rigidity.

## 1. Introduction

Since a long time, polyurethanes (PUs) have been found in a large range of applications, such as textiles, automobiles, thermal insulation, and electrical and electronics applications [1]. These polymers have excellent electrical insulation, thermal insulation, and mechanical properties; therefore, they have many versatile applications in various forms, such as foams, elastomers, coatings adhesive sealants, and electrical cable jointing.

At present, PU is mainly used for rigid or flexible foams [2]. Polyurethane foams are usually prepared by the reaction of polyols with isocyanate compounds in the presence of blowing agents. At the present time these types of foams are the largest segment in the thermosetting foam industry. A characteristic of these foams lies in the versatility of their physical properties, such as rigidness, flexibility, and viscoelasticity for a wide variety of foam densities. This versatility is derived from molecular design by the choice of raw materials and foaming reactions. The molecular weight and the functionality of polyols affect the resulting foam properties. Nevertheless, one of the main issues of these foams is their high flammability.

In many cases, fire develops in the home by spreading to upholstered furniture, bedding, furnishings and equipment, i.e., products containing PU foams. However, these different products are not subject to strong regulatory constraints in the case of Burkina Faso and their reaction to fire is often poor. Moreover, their combustion is also accompanied by the production of toxic gases, which are the most common cause of death.

Numerous works have already been carried out to improve the flame retardancy of PU foams but this field of research is still very active. Various additive FRs, especially phosphorus-based ones (in replacement of halogenated FRs), have been used alone or in combination to reduce the flammability of rigid or flexible PU foams [3,4,5,6,7,8,9]. The incorporation of FR into the mixture before foaming can modify the reactivity and the morphology (cell size) of the foams leading to a change in macroscopic properties [10]. The reactive strategy consists in using flame-retarded polyols to introduce the flame retardant into the PU macromolecules [11]. A last approach is to deposit a flame retardant coating on the surface of the PU foam after its manufacturing in order to avoid impacting mechanical properties. Yang et al. have recently reviewed this approach [12]. Among others, the layer-by-layer strategy is one of the latest promising coating approaches [13,14,15,16].

Apart from the inherent high flammability of the PU matrix, PU foams, as is the case for many other foams, have some specific issues of concern which significantly increase the fire hazard. Drysdale considers that their low thermal inertia is the main hazard, leading to easy ignition and fast flame spread [17]. Smoldering is another specific phenomenon, and it has been studied by Hadden et al. in the case of PU foams [18]. Gunther et al. recently addressed the dripping behaviour of PU foams [9]. Only a few papers investigated the phenomenon of foam collapse, which significantly increases the fire hazard. Indeed, if some foams are able to play a fire protective role (alginate foams for example) [19], many foams undergo collapse during burning, leading to the formation of a hazardous fire pool. This is especially the case for polystyrene and flexible polyurethane foams.

Kramer et al. studied these phenomena in detail for flexible polyurethane foams [20]. They explained that the collapse and melt dripping phenomena might be beneficial in terms of resistance to ignition but detrimental in terms of heat release rate and fire propagation. They showed that a thin layer of liquefied foam is formed at the surface of the foam. Then collapse starts and propagates while more liquid accumulates at the surface layer. At the end of collapse, only a fraction of material is consumed (accounting for around 29% of total heat release). Isocyanate compounds are predominantly decomposed during collapse. The foam deformation was studied and the surface recession rate corresponding to the collapse was assessed for different heat fluxes.

In their study, Kramer et al. studied the collapse of two PU foams of similar density. One was flame retarded while the second one was FR-free [20]. They measured the surface recession rate during the collapse step but they could not assess the influence of the foam density. Moreover, foam samples were relatively thin (3.9 cm); therefore, they could not estimate if the collapse rate was constant. The present article aims to provide this additional information and to confirm the surface recession rates estimated by Kramer et al. The motivation of our work is to better understand how foams collapse into hazardous pool fires and to relate the collapse mechanism to the foam microstructure.

## 2. Materials and Methods

Two virgin open-cells flexible PU foams, called LD (low density) and HD (high density) are considered in this study. They are provided by TechniMetal (Ouagadougou, Burkina Faso). They are both commonly used in upholstered furniture and mattresses. They do not contain any flame retardant.

The morphology of the samples was studied by scanning electron microscopy using an ESEM FEI Quanta 200 FEG. Elemental analysis using energy dispersive X-Ray spectroscopy (EDS—Oxford Instruments, Abingdon-on-Thames, UK) was performed to check the presence of FR elements in the samples.

Density was assessed at room temperature by weighting a specimen and measuring its dimensions. Thermal conductivity was measured by a hot wire conductivity meter FP2C from NeoTIM (Albi, France). The probe is placed between 2 samples and produces a local warm-up. The rise of temperature is measured as a function of time. The acquisition software calculates the thermal conductivity of the samples. Specific heat capacity was measured using Perkin Elmer differential scanning calorimetry (Stepscan software −2 °C/min).

Compression properties were measured using a Zwick TH010 apparatus at 10 mm/min. Sample dimensions were 100 × 100 × 100 mm^3^. Two to three samples were tested for each formulation.

Flammability was investigated using a pyrolysis combustion flow calorimeter (PCFC) (East Grinstead, UK). The sample (around 3 mg) was heated from 80 to 750 °C at 1 °C/s in a pyrolyzer under nitrogen flow and the pyrolysis gases were sent to a combustor where they were mixed with an excess of oxygen at 900 °C. In such conditions, these products were fully oxidized. The heat release rate (HRR) was calculated by the oxygen depletion method according to Huggett’s relation (1 kg of consumed oxygen corresponds to 13.1 MJ of released energy) [21]. Three samples were analysed for each foam.

Fire behaviour at bench-scale was studied using a cone calorimeter (Fire Testing Technology, East Grinstead, UK). The samples were exposed to various irradiances in well-ventilated conditions (air rate 24 L/s) in the presence of a spark igniter to force the ignition. HRR was determined according to oxygen depletion (Huggett’s relation) as measured in the Pyrolysis-Combustion Flow Calorimetry. A first series of samples of 100 × 100 × 20 mm^3^ was wrapped in aluminium foil and placed at 6 cm below a conic heater. The temperature of the upper surface was measured during test using an infrared camera (Optris). Heat flux ranged between 25 to 50 kW/m^2^.

A second series of samples of 100 × 100 × 100 mm^3^ was tested to study the collapse of foams. Samples were wrapped in aluminium foil on three sides. A digital camera (Canon) and an infrared camera (Optris) focusing on the fourth side allowed recording the foam collapse. The initial distance between the specimen and the cone was fixed to 25 mm. Heat flux was fixed to 10–35 kW/m^2^.

**Figure 1:**
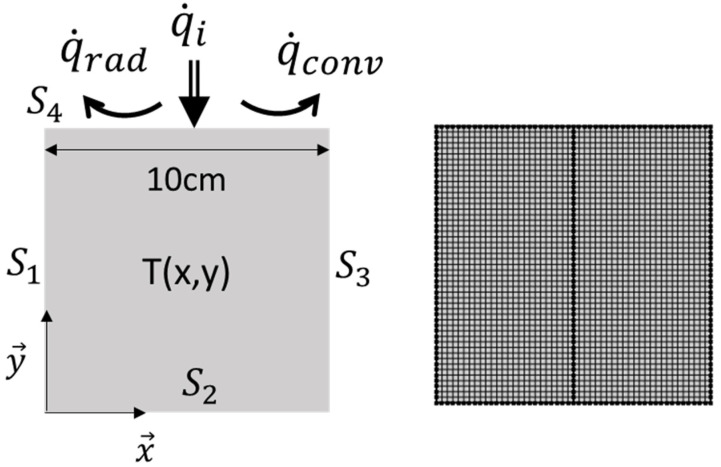
Finite element simulation: initial sample geometry, boundary conditions and mesh (${\;\dot{q}}_{i},{\;\dot{q}}_{\mathrm{rad}}{\;\mathrm{and}\;\dot{q}}_{\mathrm{conv}}$ correspond to the incident heat flux, the heat flux due to re-radiation and the heat flux due to convection losses, respectively).

Finite element simulation software (COMSOL Multiphysics) was used to model and predict local thermal variables and phenomena that can occur in a foam sample during the cone calorimeter test. Foam samples are approximated as homogeneous materials that are subjected to a heat flux applied on the upper surface. On the other surfaces, the samples are supposed to be isolated. Initial sample geometry, boundary conditions and finite elements mesh based on quadratic elements are presented in Figure 1.

The transient thermal conduction equation inside a material is given by:

(1) $$\rho C_{p}\frac{\partial T}{\partial t}-k\nabla^{2}T=q_{0}$$

where *ρ, C_p_* and *k* are the density, the specific heat and the thermal conductivity, respectively. These latest parameters are assumed to be time-independent. By considering the incident heat flux and the heat exchanges due to convection and radiation, the heat flux on the sample surface is given by:

(2) $$-k\nabla T\left(M\in S_{4},t\right)={\dot{q}}_{i}{}^{\prime \prime }\left(t\right)-a\left(T^{4}-T_{ext}^{4}\right)+h_{c}\left(T-T_{ext}\right)$$

where *a* is the product of the Stefan-Boltzmann constant (5.67 × $10^{-8}$ W/m^2^ K^4^) by the surface emissivity value (chosen equal to 0.9), $T_{ext}$ is the reference temperature (equal to 293.15 K) and *h_c_* is a constant parameter (equal to 10 W/m^2^ K) [22]

The other surfaces are assumed to be isolated:

(3) $$-k\nabla T\left(M\in S_{i},t\right)=0,\;i=1,\;2,\;3$$

## 3. Results and Discussion

### 3.1. Thermophysical Properties

**Figure 2:**
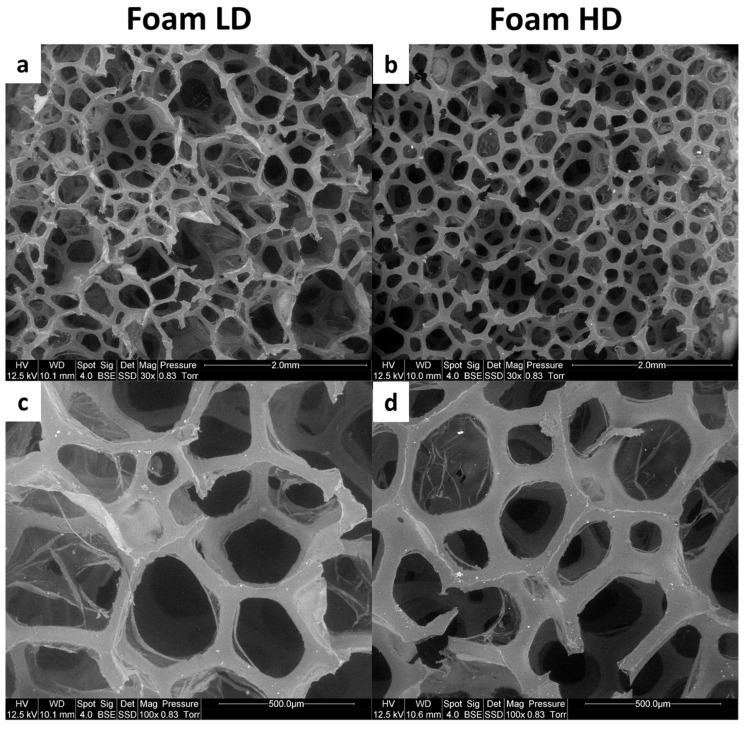
Scanning electron microscope (SEM) pictures of foams low density (LD) (**a**,**c**) and high density (HD) (**b**,**d**).

Figure 2 shows the microscopies of the foams at different magnitudes. Both foams are open cell foams. The microstructure reveals the hexagonal shape of the cells of the foam, which resembles a honeycomb, and the homogeneity of these cells. Cells of the LD foam are larger and less homogeneous. The cell walls of HD foam are slightly thicker. Analysis of elements present in the foams was carried out. EDX analysis shows that both foams have the same composition. Carbon content is about 75 wt% and oxygen content is 25 wt%. Nitrogen is not measured because its peak merges with carbon and oxygen peaks. No other peak was observed. These results show that the foams do not contain flame retardants.

Some thermophysical properties of foams are listed in Table 1. The density of both foams are in agreement with those displayed by the supplier (approximately 12 to 14 kg/m^3^ for LD foam and 22 to 24 kg/m^3^ for HD foam). Specific heat capacity was found to be 1.6 J/g.K at room temperature and 1.8 J/g.K at 100 °C, which is typical for polyurethane [23]. Heat conductivity was measured as 0.029 W/m.K for LD foam and 0.031 W/m.K for HD foam. Such values are typical of insulating materials and are one of main reasons for the high fire hazard associated to these materials. Indeed, heat cannot be transferred from the surface to the bulk due to the low heat conductivity. Therefore, surface temperature increases quickly and ignition occurs earlier.

The compression test was done to measure the resistance to compression of the foams. Compression stress ranged from 2.4 kPa at 40% of elongation for LD foam to 5 kPa at 40% for HD foam. This means that the HD foam is twice as resistant to compression.

### 3.2. Flammability at Microscale

**Figure 3:**
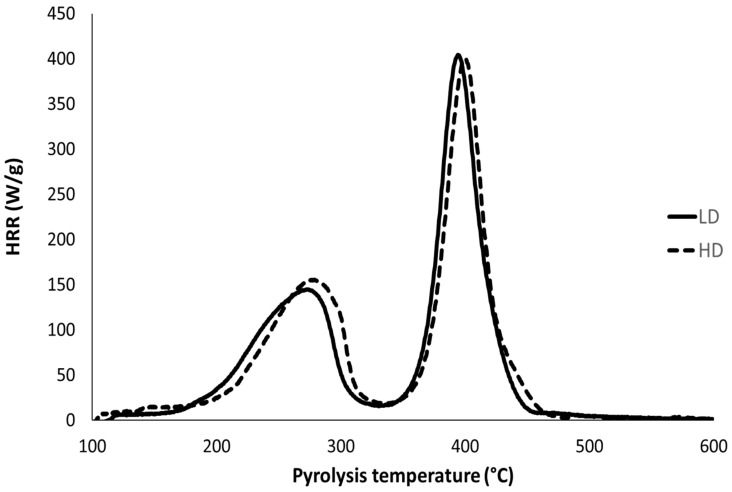
Heat release rate curves of both foams in pyrolysis–combustion flow calorimetry.

Both foams were tested using pyrolysis–combustion flow calorimetry in standard conditions (i.e., anaerobic pyrolysis up to 750 °C and complete combustion of gases released at 900 °C in an excess of oxygen). HRR curves are shown in Figure 3 and main data are listed in Table 2.

Both foams exhibit an HRR curve with two pHRRs. The first peak at 280 °C is assigned to the breaking of the urethane and urea bonds and to the decomposition of the isocyanate-based compound while the second one (which is also the main one) at 400 °C is related to polyol decomposition. The pyrolysis is complete, without residue and the heat of combustion reaches 27 kJ/g. All these features are in perfect agreement with the previous study of Kramer et al. [20]. In their study, the intensity of the peaks was threefold because the heating rate was 3 K/s (versus 1 K/s in the present work).

No significant difference can be found between the LD and HD foams. In other words, the different behaviours observed at bench scale are not related to the composition of the foams, but only to their morphology, reflected by their different densities.

### 3.3. Fire Behavior at Bench Scale

**Figure 4:**
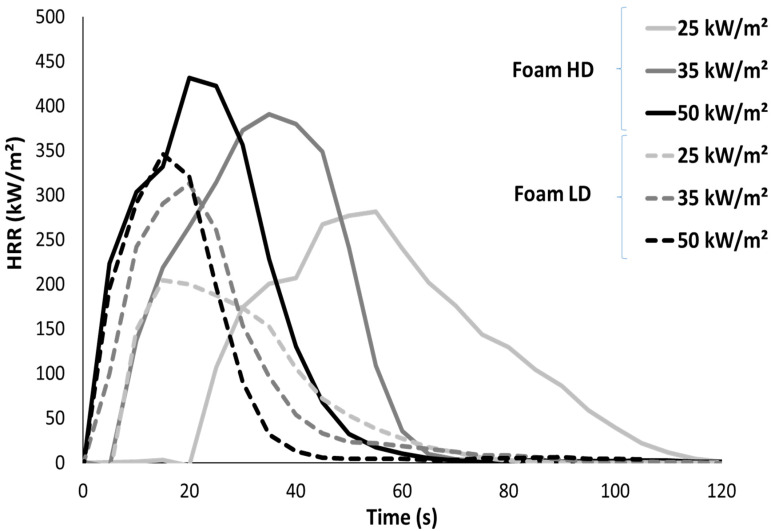
Heat release rate curves of both foams in cone calorimeter tests (sample thickness 2 cm).

Cone calorimeter tests were performed on both foams at different heat fluxes. This first series was tested on light samples (2 cm). The main results are listed in Table 3 and the HRR curves are shown in Figure 4.

HRR curves show only one pHRR reached quickly after ignition and followed by a fast HRR decrease up to extinction, especially at high heat flux; it is typical of materials exhibiting thermally thin behaviour. Indeed, amounts of the samples did not exceed 5 g.

Time-to-ignition (TTI) is very short due to low heat conductivity, as already discussed above. TTI is slightly higher for the HD foam, especially at low heat flux. Ignition occurs when the upper layer of the sample reaches a critical temperature high enough to produce fuels. The amount of energy needed is proportional to the product of the mass and specific heat (see for example typical equations for the prediction of TTI in the cone calorimeter [24]). While the HD foam is denser, the time to reach this critical temperature is also higher. These values are in agreement with those reported by Gunther et al., who have measured times-to-ignition of 8–10 s at 20 kW/m^2^ for different flexible PU foams exhibiting a density close to 30 kg/m^3^ [9]. Critical heat flux (i.e., the minimum heat flux for which the foams ignite) is very low. Indeed, even at 10 kW/m^2^ (see below for thicker foams), ignition occurs, even if the time-to-ignition is significantly higher.

The peak of the heat release rate is reached 10–15 s and 25–30 s after ignition for the LD and HD foams, respectively. Its value is in the range 270–380 kW/m^2^ and it increases with heat flux. Its value may be slightly higher for HD foam (probably because the sample mass is higher) but the difference between both foams is limited. The total heat release is around 27 kJ/g and the residue yield is negligible. Therefore the effective heat of combustion (EHC) is also close to 27 kJ/g, in good agreement with Gunther et al. [9]. The combustion efficiency is then calculated as the ratio between EHC and the heat of complete combustion measured in PCFC, and is close to 1. In other words, the combustion is complete.

**Figure 5:**
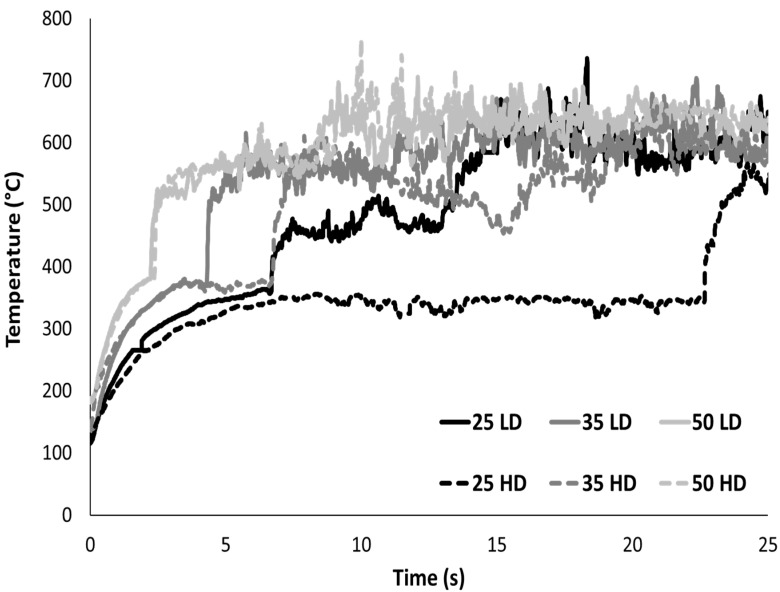
Temperature of the upper surface for both foams at different heat fluxes in cone calorimeter tests (sample thickness 2 cm).

Figure 5 shows the evolution of the temperature of the upper surface recorded by an infrared camera. Foams are positioned under the radiant cone only a few seconds before the beginning of the test and the shutter opening. Due to the heat flux irradiated from the shutter, the temperature is already high at the beginning of the test (120–160 °C). Indeed, for low conductivity materials, all the heat flux is accumulated on the surface and heat transfer from the surface to the bulk is almost negligible. Temperature increases quickly and stabilizes around 345–380 °C up to ignition. After ignition, the temperature rises rapidly and reaches a plateau around 600 °C. There is no significant difference between both foams at a fixed heat flux: the heating rate, the temperature at ignition as well as the temperature after burning are similar. Nevertheless, the delay before ignition is higher for HD foam, especially at low heat flux. As already explained, it is assigned to the higher material amount to heat after foam melting. Nevertheless, at high heat flux, the time-to-ignition is very short for both foams and no significant difference is observed between them. The dependence of the ignition temperature on the heat flux is very limited: it increases from 345–370 °C at 25 kW/m^2^ to 380 °C at 50 kW/m^2^.

**Figure 6:**
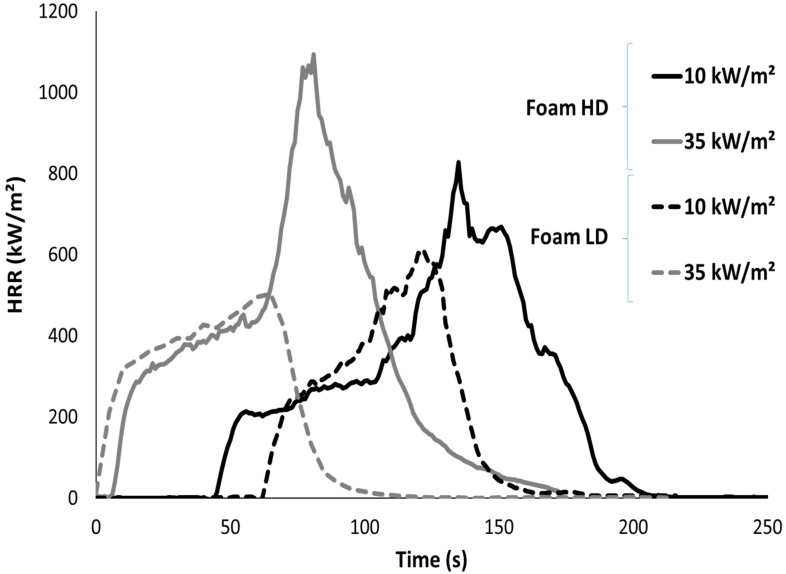
Heat release rate curves of both foams in cone calorimeter tests (sample thickness 10 cm).

Cone calorimeter tests were also performed on 10 cm-thick foams to study the foam collapse during burning. In order to easily study the collapse, the external heat flux was fixed at 10, 25 and 35 kW/m^2^. Note that, in all cases, ignition occurs before the start of the collapse. Therefore, it was not needed to ignite the samples with a small flame as performed by Kramer et al. [20]. HRR curves are shown in Figure 6 for both foams at 10 and 35 kW/m^2^. They significantly differ from the curves obtained for thin foams. Indeed, the heat release rate increases after ignition up to a pseudo-plateau. After this step a second increase to pHRR is observed. For LD foam at 35 kW/m^2^, the pseudo-plateau reaches the pHRR without sudden increase. After pHRR, the heat release rate decreases quickly due to fuel depletion up to flame out. Other researchers have already explained the main features of such curves [9,20]. The first part of the curve (a pseudo-plateau in our case, a peak in Kramer’s study) corresponds to the foam collapse. The second part for which a high pHRR is reached is related to the fire pool. Note that the peak is much higher for the HD foam because the amount of fuel in the fire pool is greater. Nevertheless, due to the removal of aluminium foil on a face of the foam, a part of the fuel flows at the end of the test (after collapse). For this reason, the calorimetric data were not analysed in detail.

**Figure 7:**
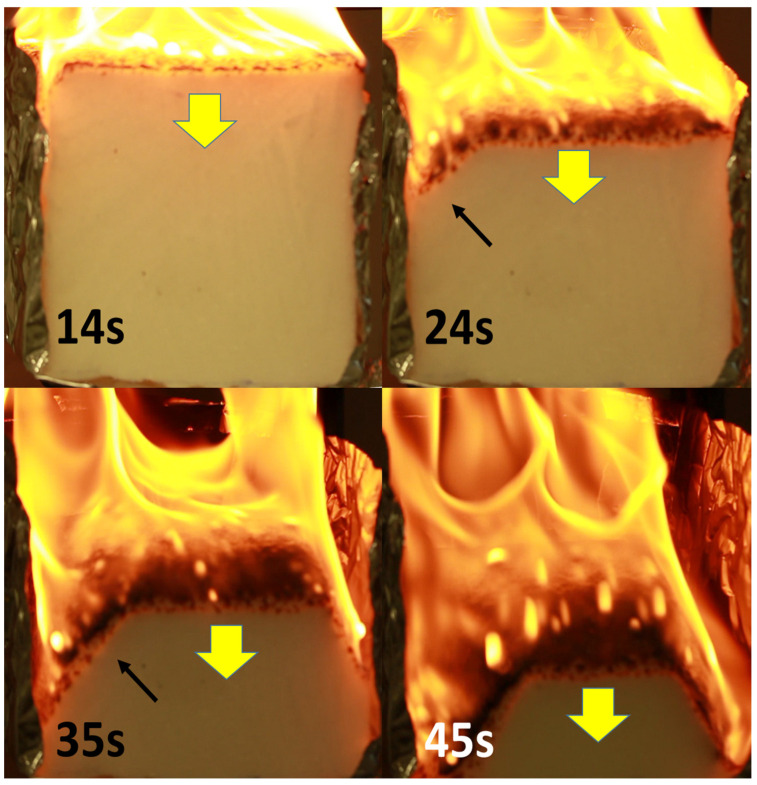
Some pictures from the digital camera for the HD foam collapse during the cone calorimeter test at 35 kW/m^2^ (sample thickness 10 cm).

Collapse was studied using a digital camera and an infrared camera. Pictures from the digital camera are shown in Figure 7 for HD foam at 35 kW/m^2^. Similar behaviour is observed for other foams and/or heat flux. As already identified by Kramer et al., a thin layer is formed at the surface constituted by melted material and black islets of char. The buoyancy of the surface is vigorous. The islets are more numerous for HD foam but they are not thermally stable. Therefore, no stable char layer is formed during the fire. At the end of the test, the residue yield is negligible. On the foam face recorded by the digital camera, the layer moves through the thickness and the layer front is parallel to the surface (yellow arrow), at least at the centre of the face. On the sides (see black arrow), the layer front progresses more quickly. It is probably due to the presence of aluminium foil. There is no close contact between the foil and the foam, and the aluminium foil may reflect the heat flux, allowing a faster heating of the foam. The collapse at the middle of the foam is slower. The following discussion is only based on the rate of front recession on the centre of the foam face (yellow arrow). Note that the rate of the front recession is constant during most of the collapse. Kramer et al. have shown that there is an almost constant ratio between the layer front rate at edge and at foam middle (ratio close to 2).

**Figure 8:**
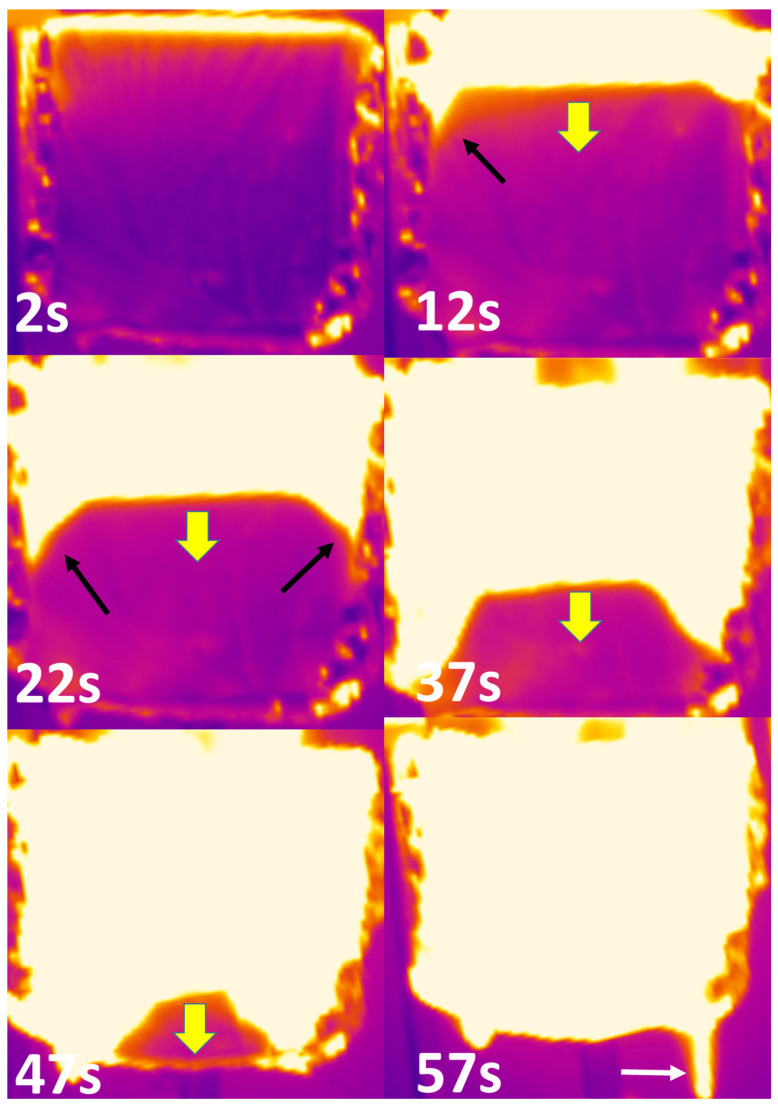
Some pictures from the IR camera for the HD foam collapse during the cone calorimeter test at 35 kW/m^2^ (sample thickness 10 cm).

Pictures from the IR camera are shown in Figure 8 for the HD foam at 35 kW/m^2^. Once again, these pictures are similar for other tests. There is a strong heat gradient and a clear border between the upper boiling material and the lower intact foam. The luminous upper part is due to the high temperature foam under burning (temperature of the upper surface reaches 600 °C while the temperature range measures by the infrared camera in this test is 0–275 °C), but also due to the aluminium foil gradually as the foam collapses. Once again the border moves through the foam thickness parallel to the surface. Black arrows show the foam sides moving at a higher rate. The white arrow highlights the flowing of the melted material at the end of the test (after collapsing).

**Figure 9:**
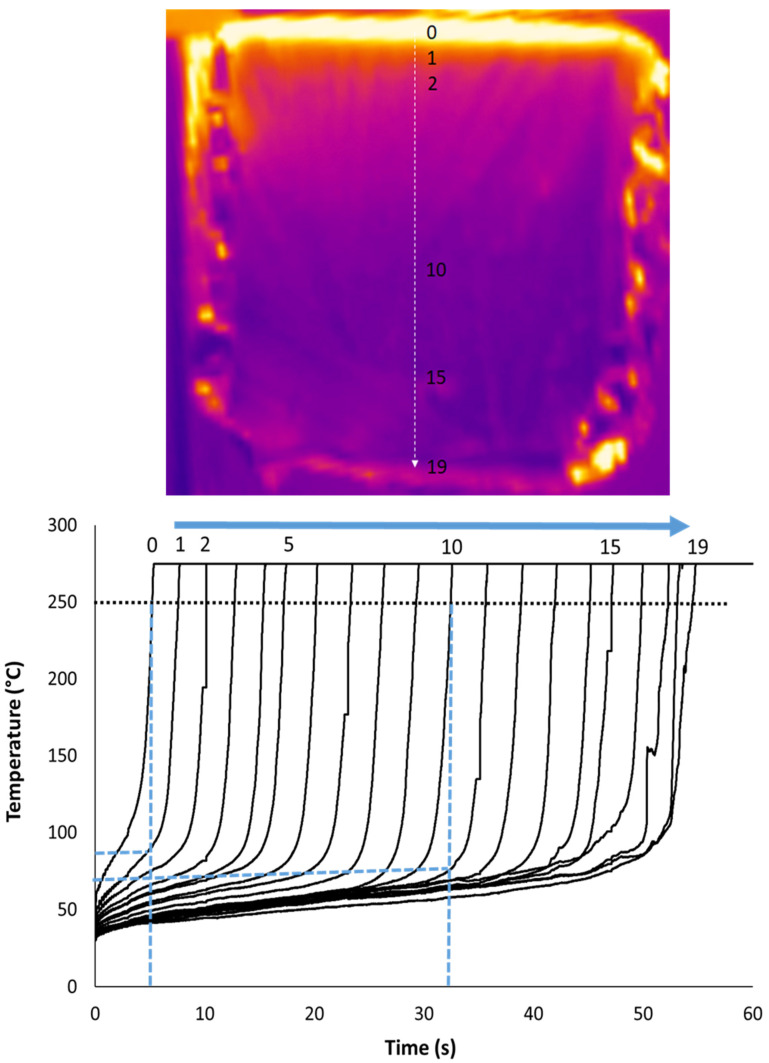
Temperature evolution through the thickness from the IR camera for the HD foam collapse during the cone calorimeter test at 35 kW/m^2^ (sample thickness 10 cm).

Temperature was recorded along a profile line through the thickness (perpendicular to the foam surface). Twenty datapoints were measured on the whole thickness of 10 cm, i.e., one point each 0.53 cm. The temperature evolution of all these 20 points is shown in Figure 9 for the HD foam tested at 35 kW/m^2^. By fixing a temperature criterion, the displacement rate of the border between the boiling matrix and the unpyrolyzed foam can be calculated. The criterion was fixed at 250 °C. This temperature is close to the temperature of the pyrolysis zone (270–330 °C) estimated by Gunther et al. [9]. However, other criteria would give the same results, taking into account the very fast increase of the temperature. Indeed, the heat gradient is very strong. When the temperature of the first point reaches 250 °C (i.e., when the extreme upper surface starts to burn), the temperature of the following point (only 0.53 cm below) is still 90 °C. When half the foam has already collapsed (i.e., when the point 10 reaches 250 °C), the point 11 just below is still at 70 °C. Gunther et al. have also observed using thermocouples that heat conduction is limited in PU foams [9].

It is obvious that the temperature at a fixed point increases very fast from the moment it exceeds 70–90 °C. This temperature range may be roughly considered for which the mechanical properties of PU drop quickly and the foam collapses. This value is close to the glass transition temperature of PU used in such foams (around 50 °C) [25].

**Figure 10:**
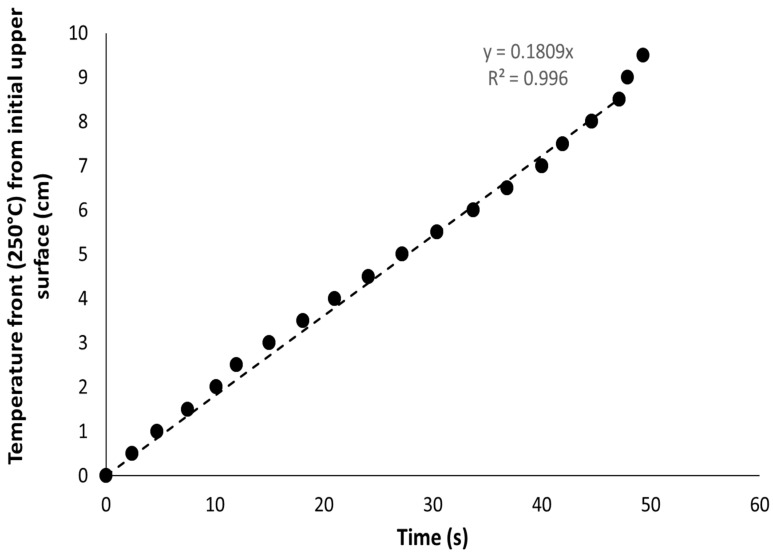
Evolution of the temperature front (measured by the IR camera) for the HD foam collapse during the cone calorimeter test at 35 kW/m^2^ (sample thickness 10 cm)—The first point was fixed arbitrarily at 0 s and 0 cm.

Figure 10 shows the temperature front (at 250 °C) versus time for the HD foam at 35 kW/m^2^. It is obvious that the front moves through the foam thickness at a constant rate except at the end of the collapse (corresponding to the 1 cm-thick bottom side of the foam).

**Figure 11:**
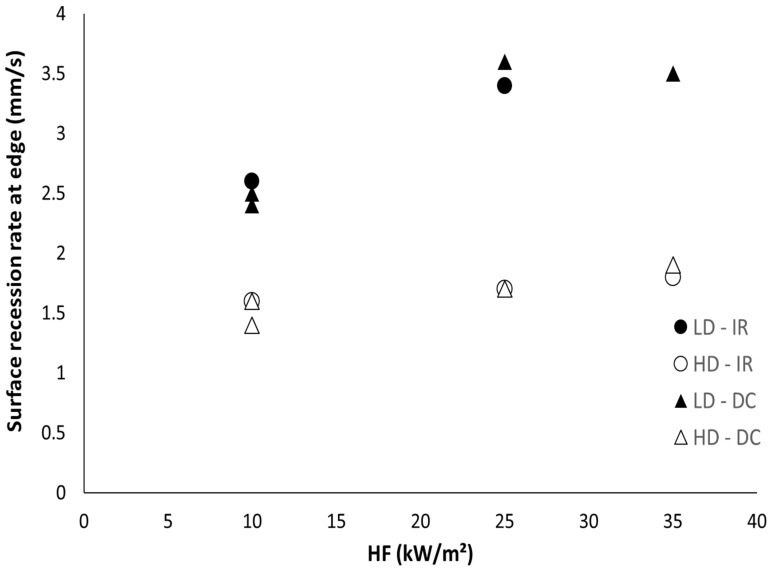
Surface recession rate at the edges for both foams at different heat fluxes, calculated from two methods.

Both methods (i.e., using digital camera and infrared camera) were applied to measure the foam collapse rate for all the foams at various heat fluxes (10, 25 and 35 kW/m^2^). The collapse rate (i.e., the surface recession rate) is similar using both methods (Figure 11). Moreover, two tests on the same foam at a fixed heat flux provide close values. The collapse rate is almost independent of the heat flux for the denser foam: around 1.6–1.7 mm/s, confirming that the heating mainly comes from the flame (and not the cone radiant), as already stated by Kramer et al.

The denser foam is very comparable to the PU foams studied by Kramer et al. which provide the collapse rate (or surface recession rate) at the middle and at the edges of the foam. Nevertheless, they provide these data only for the flame retarded foam. They observed an increase of the surface recession rate with heat flux. Their value at low heat flux (11 kW/m^2^) is in good agreement with ours: slightly lower than 1.6 mm/s. However, they measure a surface recession rate close to 2.4 mm/s at 35 kW/m^2^.

The collapse rate is significantly higher for lighter foam: 2.5–3.5 mm/s and the rate seems to increase when heat flux increases from 10 to 25 kW/m^2^. While the amount of fuel is lower in a fixed volume of LD foam, a reduced delay is needed to heat the material up to a temperature allowing its collapse. Note that heat conductivity is very low, then, the heat diffusion by conduction into the bulk is limited. The heating comes from the flame above the surface and concerns the small volume directly in contact with the flame. The heat needed to increase the temperature of this volume up to the critical value corresponding to its collapse is directly related to the product of its heat capacity and its mass. Therefore, the ratio between the collapse rate *V* between LD and HD should be equal to the reciprocal ratio between their density ρ (taking into account that the heat capacity is the same for both foams).

(4) $$\frac{V_{HD}}{V_{LD}}≅\frac{\rho_{LD}}{\rho_{HD}}$$

According to the heat flux, $\frac{V_{HD}}{V_{LD}}$ is in the range 0.49–0.67 while $\frac{\rho_{LD}}{\rho_{HD}}$ is 0.58. This confirms that the collapse rate is mainly dependent on the foam density.

Interestingly, the relative mass loss at the end of the collapse step is similar for both foams (around 25%). Kramer et al. also observed that only 29% of total heat was released at the end of collapse. It means that only a minor fraction of the material was effectively burnt during collapse. The main fraction was just liquefied and burnt during the second step. Once again, this fraction is minimally dependent on heat flux, evidencing that the burning is piloted by the flux coming from the flame. This fraction is also similar for both foams, i.e., the fuel amount released in the gas phase is much higher for the HD foam. In fact, during the collapse, mainly isocyanate compounds are burnt. Indeed, their decomposition temperature is low: 280 °C versus 400 °C for the polyol part according to the PCFC data. It may be assumed that the heating during collapse leads to the decomposition of isocyanate but not polyol in the liquefied layer. This is in agreement with the temperature of pyrolysis zone estimated by Gunther et al. [9]: 270–330 °C (even if the extreme surface is at around 600 °C).

Of course, while 75% of the material has not been burnt yet at the end of collapse, the amount of fuel during the pool fire step is much higher for denser foam and explains why the second pHRR is very high for this foam.

### 3.4. Preliminary Simulations

**Figure 12:**
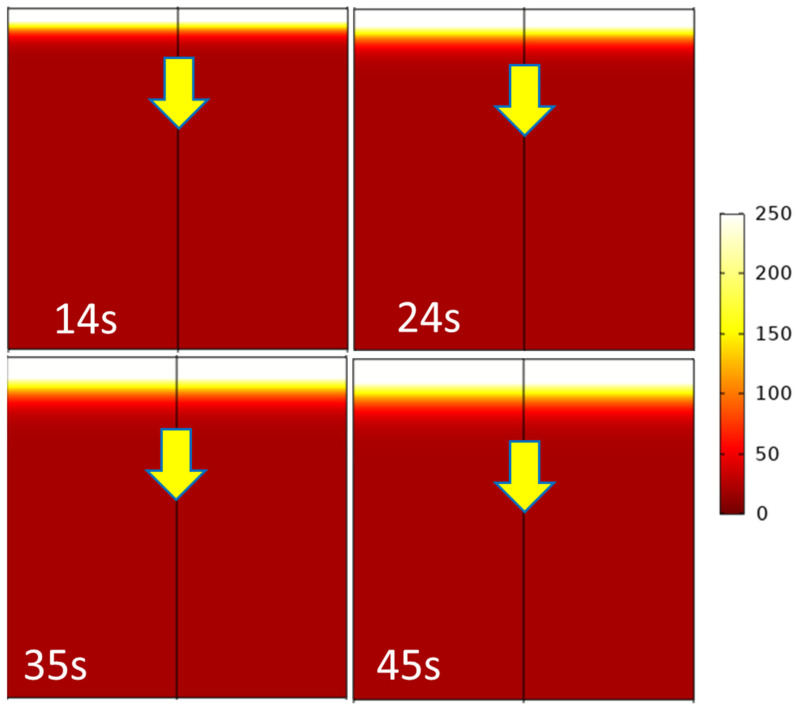
Simulation of the surface thermal properties in the range of 0 to 250 °C.

**Figure 13:**
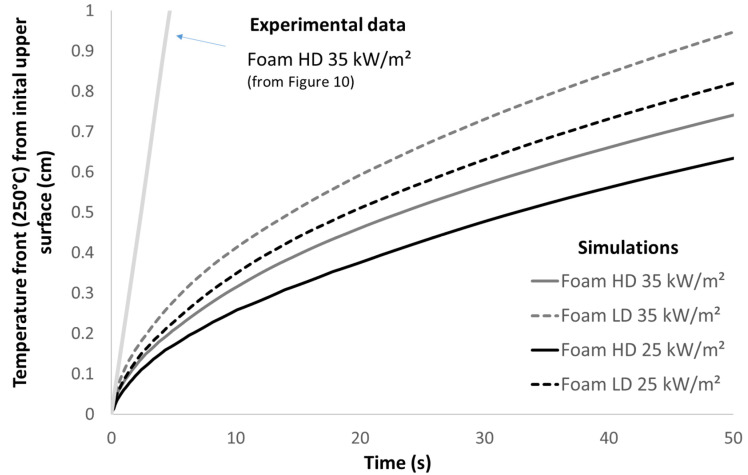
Evolution of the temperature front versus time for different simulations and experimental data corresponding to the HD foam at 35 kW/m^2^ (Figure 10).

As explained in the Materials and Methods Section, preliminary simulations considering equivalent homogeneous materials were carried out. A contour map of the surface temperatures for the *HD* foam under the heat flux of 35 kW/m^2^ is given in Figure 12 (values being capped in the range of 0 to 250 °C). This temperature (250 °C) was chosen to allow a direct comparison with the experimental cartographies (Figure 8). Keep in mind that these cartographies allow following the collapse, because the experimental temperature front at 250 °C is correlated to the surface recession rate. As expected, simulation results plotted in Figure 13 confirm that the thermal conduction rate increases with a higher heat flux or lower foam density, this latter parameter being the most important one.

However, the predicted evolution of the temperature front (at 250 °C) in Figure 12 and Figure 13 is much slower than the experimental observations displayed in Figure 8 and Figure 10 or Figure 11. Obviously, the simulations do not perfectly fit the experimental conditions. For example, the edges are not fully isolated as assumed in the numerical analyses. After ignition, heat flux from the flame contributes to the heating. Nevertheless, even if considering a much higher heat flux, the simulated temperature front rate remains very low. Moreover, Figure 10 shows that the temperature front moves through the thickness at a constant rate. On the contrary, the simulated temperature front rate decreases progressively. Indeed, the heat diffusion slows down due to the insulating PU foam as the temperature front progresses. This is not the case from an experimental point of view, due to foam collapse.

The comparison between the simulation results and the experimental observations leads to the conclusion that the thermal conduction simulation of a macrohomogeneous material with appropriate material values for the foam was unable to predict the correct evolution of the temperature front associated with the local collapse. This leads to assume that microstructural features of the foam should be involved in the observed phenomenon. Buckling of the foams cell walls due to local loss of stiffness induced by temperature increase could occur and drive the foam collapse at a macroscopic level. Further numerical analyses are needed to clarify this point.

## 4. Conclusions

Two open-cell flexible PU foams were tested in cone calorimeter tests to assess the influence of their density, especially on the collapse step, in order to complete the pioneering work from Kramer et al. Both foams differ only by their cells’ morphology and therefore from their density and related properties (heat conductivity, compression stress). They are free of flame retardants and their decomposition at microscale is the same with two characteristic peaks of heat release rate.

In the cone calorimeter, thin foams exhibit fast ignition (due to the low heat conductivity) and only one pHRR in the range 280–380 kW/m^2^. Its value is slightly higher for denser foam due to the higher amount of PU. The temperature at ignition is around 360 (±20) °C for both foams and the surface temperature during burning does not exceed 600–650 °C.

Decomposition occurs in two steps for 10 cm-thick foams: the first one is the collapse corresponding to a pseudo-plateau of the HRR. After collapse, the pool fire leads to a very intense pHRR. The collapse step lasts several dozens of seconds (less than 1 min).

The surface recession rate was measured using two methods (namely digital and infrared cameras) on thick foams. The digital camera recorded the displacement of the thin liquid layer while the infrared camera recorded the displacement of the temperature front (fixed at 250 °C here, close to the estimated temperature of the pyrolysis zone). There is a strong heat gradient between the pyrolysis zone and the underlying material due to the low heat conductivity of the foams. Despite the foam deformation during the burning, the collapse front is found to be parallel to the surface on the centre of the sample face, allowing a proper measurement. Both methods provide similar values for the surface recession rate.

The surface recession rate was found to be constant throughout the whole collapse step, i.e., through the 10 cm thickness of the foam, evidencing that the heat flux piloting the collapse comes from the flame. The rate was found to be minimally dependent on the heat flux, ranging from 10 to 35 kW/m^2^. Its value is close (1.7 to 2.0 mm/s) to that found by Kramer et al. for equivalent density (24 kg/m^3^). The rate is higher for the lighter foam (2.5–3.8 mm/s) and shows a slightly higher dependence on heat flux (between 10 to 25 kW/m^2^). The ratio between the surface recession rates is more or less inversely proportional to the ratio between the densities. According to this relation, it should be possible to estimate the collapse rate for a larger range of density: for example, a foam with a density of 100 kg/m^3^ would collapse roughly at a rate of 0.41–0.48 mm/s, leading to a longer period of collapse (around 3 min for a 10 cm-thick foam).

The same material fraction (around 25%, corresponding mainly to the isocyanate part of the foams) is burnt during collapse for both foams. Therefore, a high density promotes a lower collapse rate but a more intense pool fire (during which the polyol fraction is burnt).

Simple numerical simulations on equivalent homogeneous materials lead to very slow surface recession (at least ten times slower) in comparison to the experimental ones. The local buckling of the cell foams should be considered to predict the foam collapse. Further investigations are needed to clarify this point.

## Figures and Tables

**Table 1:** Foam thermal properties.

	Foam LD	Foam HD
Density (kg/m^3^)	14	24
Thermal conductivity (W/m·K)	0.029	0.031
Specific heat capacity (J/g·K)	1.6	
Compression stress at 40% of elongation (kPa)	2.4	5.0

**Table 2:** Main data from pyrolysis–combustion flow calorimetry on both foams.

Foam	pHRR1 (W/g)	T pHRR1 (°C)	pHRR2 (W/g)	T pHRR2 (°C)	THR (kJ/g)	Residue Fraction	Heat of Combustion (kJ/g)
LD foam	121	278	416	398	26.6	0.01	26.8
HD foam	135	282	420	404	27.1	0.01	27.3

**Table 3:** Main data from cone calorimeter tests on 2 cm-thick foams.

Foam	Heat Flux (kW/m^2^)	TTI (s)	pHRR (kW/m^2^)	TpHRR (s)	THR (kJ/g)
LD	25	8	279	21	27.3
LD	35	5	297	16	28.6
LD	50	2	355	18	28.2
HD	25	14	276	41	25.6
HD	35	7	357	29	27.8
HD	50	2	381	26	28.6

## References

[1] Saunders J.H., Frisch K.C. (1964). Polyurethanes Chemistry and Technology, Part II: Technology.

[2] Gama N., Ferreira A., Barros-Timmons A. (2018). Polyurethane Foams: Past, Present, and Future. Materials.

[3] Członka S., Strąkowska A., Strzelec K., KAIRYTĖ A., Kremensas A. (2020). Melamine, silica, and ionic liquid as a novel flame retardant for rigid polyurethane foams with enhanced flame retardancy and mechanical properties. Polym. Test..

[4] Acuña P., Lin X., Calvo M.S., Shao Z., Pérez N., Villafañe F., Rodríguez-Pérez M.Á., Wang D.-Y. (2020). Synergistic effect of expandable graphite and phenylphosphonic-aniline salt on flame retardancy of rigid polyurethane foam. Polym. Degrad. Stab..

[5] Mohammadi A., Wang D.-Y., Hosseini A.S., De La Vega J. (2019). Effect of intercalation of layered double hydroxides with sulfonate-containing calix[4]arenes on the flame retardancy of castor oil-based flexible polyurethane foams. Polym. Test..

[6] Chang C., Liu L., Li P., Xu G., Xu C. (2020). Preparation of flame retardant polyurethane foam from crude glycerol based liquefaction of wheat straw. Ind. Crop. Prod..

[7] Neisius M., Liang S., Mispreuve H., Gaan S. (2013). Phosphoramidate-Containing Flame-Retardant Flexible Polyurethane Foams. Ind. Eng. Chem. Res..

[8] Zeng S.-L., Xing C.-Y., Chen L., Xu L., Li B.-J., Zhang S. (2020). Green flame-retardant flexible polyurethane foam based on cyclodextrin. Polym. Degrad. Stab..

[9] Günther M., Levchik S.V., Schartel B. (2020). Bubbles and collapses: Fire phenomena of flame-retarded flexible polyurethane foams. Polym. Adv. Technol..

[10] Kuranska M., Beneš H., Sałasinska K., Prociak A., Malewska E., Polaczek K. (2020). Development and Characterization of “Green Open-Cell Polyurethane Foams” with Reduced Flammability. Materials.

[11] Wu N., Niu F., Lang W., Yu J., Fu G. (2019). Synthesis of reactive phenylphosphoryl glycol ether oligomer and improved flame retardancy and mechanical property of modified rigid polyurethane foams. Mater. Des..

[12] Yang H., Yu B., Song P., Maluk C., Wang H. (2019). Surface-coating engineering for flame retardant flexible polyurethane foams: A critical review. Compos. Part B Eng..

[13] Mu X., Yuan B., Pan Y., Feng X., Duan L., Zong R., Hu Y. (2017). A single α-cobalt hydroxide/sodium alginate bilayer layer-by-layer assembly for conferring flame retardancy to flexible polyurethane foams. Mater. Chem. Phys..

[14] Kim Y.S., Harris R., Davis R. (2012). Innovative Approach to Rapid Growth of Highly Clay-Filled Coatings on Porous Polyurethane Foam. ACS Macro Lett..

[15] Carosio F., Ghanadpour M., Alongi J., Wågberg L. (2018). Layer-by-layer-assembled chitosan/phosphorylated cellulose nanofibrils as a bio-based and flame protecting nano-exoskeleton on PU foams. Carbohydr. Polym..

[16] Carosio F., Alongi J. (2016). Ultra-Fast Layer-by-Layer Approach for Depositing Flame Retardant Coatings on Flexible PU Foams within Seconds. ACS Appl. Mater. Interfaces.

[17] Drysdale D.D. (1987). Fundamentals of the Fire Behaviour of Cellular Polymers. Fire and Cellular Polymers.

[18] Hadden R., Alkatib A., Rein G., Torero J.L. (2014). Radiant Ignition of Polyurethane Foam: The Effect of Sample Size. Fire Technol..

[19] Vincent T., Vincent C., Dumazert L., Otazaghine B., Sonnier R., Guibal E. (2020). Fire behavior of innovative alginate foams. Carbohydr. Polym..

[20] Krämer R., Zammarano M., Linteris G., Gedde U., Gilman J. (2010). Heat release and structural collapse of flexible polyurethane foam. Polym. Degrad. Stab..

[21] Huggett C. (1980). Estimation of rate of heat release by means of oxygen consumption measurements. Fire Mater..

[22] Hopkins D., Quintiere J. (1996). Material fire properties and predictions for thermoplastics. Fire Saf. J..

[23] Zaikov G.E., Polishchuk A.Y. (1994). Polymer Flammability. Int. J. Polym. Mater..

[24] Schartel B., Hull T.R. (2007). Development of fire-retarded materials—Interpretation of cone calorimeter data. Fire Mater..

[25] Duquesne S. (2001). Etude des Procédés d’Ignifugation de Substrats Polymères par Revêtements Intumescents Application aux Polyuréthanes. PhD. Thesis.

