# Correction: Sonnier, R., et al. Influence of Density on Foam Collapse under Burning. *Polymers* 2020, *13*, 13

**DOI:** 10.3390/polym13040554

**Published:** 2021-02-13

**Authors:** Abdoul Fayçal Baguian, Salifou Koucka Ouiminga, Claire Longuet, Anne-Sophie Caro-Bretelle, Stéphane Corn, Antoine Bere, Rodolphe Sonnier

**Affiliations:** 1 Laboratoire de Physique et de Chimie de l’Environnement, Université Joseph KI-ZERBO, Ouagadougou 03 BP 7021, Burkina Faso; bag_fay@yahoo.fr (A.F.B.); salif0477@yahoo.com (S.K.O.); berebiya@yahoo.fr (A.B.); 2 IMT—Mines Ales, Polymers Hybrids and Composites (PCH), 6 Avenue de Clavières, F-30319 Alès CEDEX, France; claire.longuet@mines-ales.fr; 3 LMGC, IMT Mines Ales, Université Montpellier, CNRS, F-30319 Alès CEDEX, France; anne-sophie.caro@mines-ales.fr (A.-S.C.-B.); stephane.corn@mines-ales.fr (S.C.)

The authors wish to make the following corrections to this paper [1]: in the original version of our article, the sentence “this value is close to the glass transition temperature of PU used in such foams (around 50 °C) [25]” is scientifically incorrect. The glass transition temperature indicated in the sentence (50 °C) corresponds to rigid foams rather than to flexible foams. We apologize for the original error. To correct this oversight, the sentence and reference [25] should be removed from the paper.

The authors apologize for any inconvenience caused and state that other scientific information is unaffected. The original article has been updated.
